# Expression of Prostaglandin E_2_ Enzymes in the Synovium of Arthralgia Patients at Risk of Developing Rheumatoid Arthritis and in Early Arthritis Patients

**DOI:** 10.1371/journal.pone.0133669

**Published:** 2015-07-30

**Authors:** Maria J. H. de Hair, Patrick Leclerc, Elize C. Newsum, Karen I. Maijer, Marleen G. H. van de Sande, Tamara H. Ramwadhdoebe, Dirkjan van Schaardenburg, Lisa G. M. van Baarsen, Marina Korotkova, Danielle M. Gerlag, Paul-Peter Tak, Per-Johan Jakobsson

**Affiliations:** 1 Division of Clinical Immunology and Rheumatology, Academic Medical Center/University of Amsterdam, Amsterdam, the Netherlands; 2 Rheumatology research Unit, Department of Medicine, Karolinska Institute, Stockholm, Sweden; 3 Department of Experimental Immunology, Academic Medical Center/University of Amsterdam, Amsterdam, the Netherlands; 4 Department of Rheumatology, Reade, Amsterdam, The Netherlands; Faculté de médecine de Nantes, FRANCE

## Abstract

**Objective:**

Arthralgia may precede the development of synovial inflammation in autoantibody-positive individuals at risk of developing rheumatoid arthritis (RA). A major pathway involved in pain is the prostaglandin (PG) E_2_ pathway. We investigated this pathway in the synovium of individuals with RA-specific autoantibodies and in early arthritis patients.

**Methods:**

Nineteen autoantibody-positive individuals (IgM-rheumatoid factor and/or anti-cyclic citrullinated peptide antibodies) with arthralgia (n=15) and/or a positive family history of RA (n=8), who had been prospectively followed for at least 2 years, were included. In addition, we included early arthritis patients (disease-modifying antirheumatic drug naïve) who after 2 years follow up fulfilled classification criteria for RA (n=63), spondyloarthritis (SpA; n=14), or had unclassified arthritis (UA; n=27). In all subjects we assessed pain and performed synovial biopsy sampling by mini-arthroscopy at baseline. Tissue sections were examined by immunohistochemistry to detect and quantify PGE_2_ pathway enzymes expression levels (mPGES-1; COX-1 and -2; 15-PGDH).

**Results:**

In both study groups synovial expression of PGE_2_ enzymes was not clearly related to pain sensation. Expression levels at baseline were not associated with the development of arthritis after follow up (6 out of 19 autoantibody-positive individuals). However, in early SpA patients the expression levels of mPGES-1 and COX-1 were significantly increased compared to RA and UA patients.

**Conclusion:**

Pain in autoantibody-positive individuals without synovial inflammation who are at risk of developing RA and in early arthritis patients may be regulated by pathways other than the PGE_2_ pathway or originate at sites other than the synovium. In contrast, in SpA, the PGE2 pathway may be inherently linked to the pathophysiology/etiology of the disease.

## Introduction

Rheumatoid arthritis (RA) is an inflammatory autoimmune disease characterized by synovial inflammation with clinically apparent arthritis, which may lead to joint destruction. Joint pain is a major burden for RA patients and it is important to characterize the underlying molecular mechanisms.

The RA-specific autoantibodies rheumatoid factor (RF) and anti-citrullinated peptide antibodies (ACPA) can be present years before onset of clinical disease [1–3]. Their presence is associated with an increased risk of developing RA [4,5], enabling the study of the preclinical phase of RA. The main target tissue in established RA is the synovium, which is characterized by hyperplasia of the intimal lining layer and accumulation of inflammatory cells in the synovial sublining [6]. Synovial cellular infiltration occurs relatively late in the disease process, most likely not more than a few weeks to months before the development of clinically manifest arthritis [7,8]. During the preclinical phase, patients may have arthralgia, even before inflammatory cells infiltrate the synovial tissue [7,8]. It is currently not clear what the underlying mechanisms are explaining pain in the absence of a synovial inflammatory cell infiltrate in subjects at risk of developing RA. Prostaglandin E_2_ (PGE_2_) may play an important role, as the prostaglandin pathway has a major involvement in arthritic pain, which is illustrated by the beneficial effects of nonsteroidal anti-inflammatory drugs (NSAIDs) and COX-2 inhibitors (COXibs). PGE_2_ is a powerful inflammatory mediator that can act both locally and centrally to mediate hyperalgesia. Locally, in the synovial tissue, unmyelinated nerve fibers may be sensitised by PGE_2_ upon inflammatory stimuli [9]. To produce PGE_2_, arachidonic acid is metabolized into prostaglandin H_2_ (PGH_2_) by the enzymes cyclooxygenase-1 (COX-1) or COX-2. COX-1 is constitutively expressed by a variety of cells and tissues and contributes to homeostasis. COX-2 expression, on the other hand, is almost absent under normal conditions but increases following pro-inflammatory stimuli. The PGH_2_ yield is subsequently transformed into PGE_2_ by one of three prostaglandin E synthases (PGES) of which microsomal PGES-1 (mPGES-1) plays a predominant role under inflammatory conditions [10,11]. PGE_2_ levels are further regulated at the catabolic end by the enzyme 15-hydroxy prostaglandin dehydrogenase (15-PGDH). With regard to the relationship between the levels of PGE_2_ enzymes, NSAIDs and the PGE_2_ biosynthesis it is known that glucocorticoids can downregulate the enzymes of the induced PGE2 pathway, while NSAIDs and COXibs rather inhibit the enzymatic catalysis of PGE2 [12].

Genetic deletion studies have highlighted the importance of COX and mPGES-1 in different mouse models of arthritis, reducing incidence, severity and pain in knock-out mice under the relevant experimental settings [13–15]. Moreover, PGE_2_ pathway enzymes are expressed both at the mRNA and protein level in the inflamed synovium of RA, spondyloarthritis (SpA) and osteoarthritis (OA) patients and they are especially high in ankylosing spondylitis patients [16–18]. This indicates that there are differences in this pathway related to the type of disease.

We hypothesized that synovial expression of enzymes of the PGE_2_ pathway was positively related to arthralgia and contributed to the development of arthritis in autoantibody-positive arthralgia patients at risk of developing RA. These analyses will give insight into the involvement of the prostaglandin pathway in arthralgia and the development of RA and thereby shed light on the relevance of targeting this pathway in the preclinical phase of the disease. In addition, we studied the expression of these enzymes in the synovial tissue of early arthritis patients with different diseases as well as in prognostic subgroups to get insight into the contribution of the prostaglandin pathway in specific diseases or to the persistence of the disease. These analyses may help to identify subgroups of early arthritis patients in which the use of NSAIDs and COXibs may be more effective. We focused on expression of these enzymes in the synovial tissue, as the synovium is the main target tissue in arthritis. Overall, our study may help to develop NSAIDs/COXibs with higher efficacy, possibly by developing drugs that target more than one arm of the eicosanoid pathway.

## Materials and Methods

### Study subjects

Two groups were included. First, individuals with arthralgia and/or a positive family history of RA, but without any evidence of arthritis after thorough physical examination, have been recruited since June 2005 [7,8] and followed over time to assess arthritis onset. They are all positive for IgM-RF and/or ACPA. For this study, we selected 19 individuals who had been followed for at least 2 years: 15 individuals with arthralgia and 4 individuals without arthralgia, defined by absence of pain on joint examination and a score on a visual analogue scale (VAS) for pain (scale 0–100 mm) of 0. These individuals are collectively referred to as ‘autoantibody-positive individuals’.

The second group consisted of early arthritis patients (arthritis duration < one year, disease-modifying antirheumatic drug (DMARD) naïve) with an inflamed wrist, knee or ankle joint who were included in the early arthritis cohort of the Academic Medical Center (AMC) in Amsterdam [19], since August 2002. To analyse PGE_2_ pathway enzymes expression exclusively in patients having a definite diagnostic classification, we selected all patients from this cohort who, after 2 years of follow-up, fulfilled either the 2010 ACR/EULAR criteria for RA (n = 63), or the European Spondyloarthritis Study Group criteria [20] for SpA (n = 14) or who did not meet classification criteria for any established rheumatic disease and were classified as unclassified arthritis (UA, n = 27). Only patients for whom synovial tissue slides were available for immunohistochemistry, based on our quality control system, were included.

### Ethics Statement

The study was performed according to the principles of the Declaration of Helsinki, approved by the institutional review board of the Academic Medical Center, Amsterdam, the Netherlands, and all study subjects gave written informed consent.

### Study design

At baseline and yearly study visits, the following clinical and laboratory parameters were obtained: patient’s visual analogue scale (VAS) for global disease activity (scale 0–100 mm); patient’s VAS for pain in general; patient’s VAS for pain in the biopsied joint; 68 tender joint count (68TJC) and 66 swollen joint count (66SJC); morning stiffness in minutes; IgM-RF levels using IgM-RF ELISA (Sanquin, Amsterdam, the Netherlands (upper limit of normal (ULN) 12.5 IU/mL until December 2009 and thereafter using IgM-RF ELISA (Hycor Biomedical, Indianapolis, IN (ULN 49 IU/mL)); ACPA using anti-citrullinated cyclic peptide (CCP)2 ELISA CCPlus (Eurodiagnostica, Nijmegen, the Netherlands (ULN 25 kAU/L)); erythrocyte sedimentation rate (ESR); serum levels of C-reactive protein (CRP); x-rays of hands and feet.

In the group of autoantibody-positive individuals an additional study visit was performed for individuals who developed arthritis at which the presence of arthritis was independently assessed by two investigators (MS and DG or MH and DG).

Early arthritis patients were followed for 2 years, after which they were classified for arthritis outcome: self-limiting disease, defined as no arthritis on examination and no use of DMARDs or steroids in the preceding three months, persistent non-erosive disease, defined as presence of arthritis in at least 1 joint and/or of DMARDs or steroids in the preceding three months, or persistent erosive disease, defined as presence of joint erosions on radiographs of the hands and/or feet [21].

### Synovial biopsy sampling

At baseline, all study subjects underwent arthroscopic synovial biopsy sampling as previously described [22,23]. In the autoantibody-positive individuals synovial biopsy sampling was performed in a knee joint in all cases and in the early arthritis patients in an inflamed wrist, knee or ankle joint. Six to 8 synovial biopsy samples were collected for immunohistochemistry (IHC) to correct for sampling error, as described previously [24]. The synovial biopsy samples were snap-frozen *en bloc* in Tissue-Tek OCT (Miles, Elkhart, IN) immediately after collection. Sections (5 μm each) were cut and mounted on Star Frost adhesive glass slides (Knittelgläser, Braunschweig, Germany). Sealed slides were stored at -80°C until further use.

### Immunohistochemistry and analysis

Synovial tissue sections were fixed in 2% formaldehyde and immunohistochemical staining was performed using rabbit polyclonal antiserum raised towards mPGES-1 [18], rabbit polyclonal anti-COX-1 (Cayman Chemical, Ann Harbor, MI) and mouse monoclonal anti-COX-2 (CX229; Cayman Chemical). Synovial tissue sections of the autoantibody-positive individuals were additionally stained using rabbit polyclonal anti-15-PGDH (Novus Biologicals, Littleton, CO). Staining was performed using a 2 step immunoperoxidase method as previously described [25]. As negative control, isotype-matched immunoglobulins were applied to the sections instead of the primary antibody. Staining of the synovial tissue sections of autoantibody-positive individuals was developed using diaminobenzidine (DAB; Vector laboratories; Burlingame, CA) and quantified by computer-based image analysis using a Leica DM RXA2 microscope and the Leica Qwin pro software. Results were expressed as percentage of positive stained area per total tissue area. Staining of the synovial tissue of early arthritis patients was developed using 3-amino-9-ethylcarbazole (AEC; Vector Laboratories) and expression of the markers was quantified using digital image analysis as described previously [26,27]. Expression levels are presented as integrated optical density (IOD)/mm^2^, an arbitrary unit representing the intensity of staining per mm^2^ [28]. These experiments were performed in four separate sessions; diagnoses were randomized over the sessions. To correct for between-session variation, the factor correction program was used [29]. Previously, in a subset of the early arthritis patients synovial tissue sections were stained using mouse monoclonal anti-CD68 to detect macrophages, anti-CD3 for T cells and anti-CD55 for fibroblast like synoviocytes (FLS), as described before [30].

### Statistical analysis

Continuous, normally distributed data were presented as mean (standard deviation, SD) and differences between study groups were analyzed using ANOVA. Not normally distributed data were depicted as median (interquartile range, IQR) and differences between study groups were analyzed using Kruskal-Wallis and Mann Whitney tests where appropriate. Categorical data were depicted as number (%) and differences between study groups analyzed using chi2 test. Synovial markers in early arthritis patients, analysed by digital image analysis, were log transformed after which these data were normally distributed. Differences between patients who did or did not use NSAIDs were analysed using the Student’s t-test. Differences between diagnostic groups were analysed using analysis of variance (ANOVA) and Tukey’s post-hoc test. Bivariate correlations between synovial tissue markers and clinical disease parameters were analyzed using either Pearson or Spearman rank correlation test. Statistical analysis was performed using PASW Statistics 18 (SPSS Inc, Chicago, IL).

## Results and Discussion

### Autoantibody-positive individuals

Nineteen autoantibody-positive individuals were included in this study. Baseline characteristics of autoantibody-positive individuals are depicted in Table 1.

**Table 1:** Baseline characteristics of autoantibody-positive individuals.

	N = 19
Sex, female (n (%))	10 (53)
Positive family history of RA (n (%))	8 (21)
Age, years (median (IQR))	48 (43–54)
IgM-RF positive (n (%))	12 (63)
ACPA positive (n (%))	14 (74)
IgM-RF and ACPA double pos (n (%))	7 (37)
ESR, mm/hr (median (IQR)	8 (3–19)
CRP, mg/L (median (IQR)	2.2 (1.2–6.2)
Morning stiffness, minutes (median (IQR)	5 (0–15)
Arthralgia (n (%))	15 (79)
Arthralgia in the biopsied knee joint (n (%)) *	7 (47)
VAS pain, mm (median (IQR)) *	29 (6–57)
VAS disease activity, mm (median (IQR))	12 (1–40)
68 TJC (n) *	1 (0–2)
66 SJC (n)	0
NSAID use (n (%))	4 (21)

IgM-RF = IgM rheumatoid factor; ACPA = anti-citrullinated protein antibodies; ESR = erythrocyte sedimentation rate; CRP = C-reactive protein; VAS = visual analogue scale (rang 0–100 mm); 68 TJC = tender joint count of 68 joints; 66 SJC = swollen joint count of 66 joints;

* Only in individuals with arthralgia;

IQR: interquartile range; NSAID: non steroidal anti-inflammatory drug

#### Synovial expression of PGE_2_ enzymes is not clearly related to arthralgia and development of arthritis in autoantibody-positive individuals

Synovial expression of PGE_2_ pathway enzymes was not lower in the group of individuals who used NSAIDs (n = 4; mPGES-1 median 0.40, range 0.18–1.05; COX-1 median 1.85, range 0.84–2.45; COX-2 median 0.29, range 0.16–0.54; 15-PGDH median 0.11, range 0.07–0.18) compared to those who did not (n = 15; mPGES-1 median 0.54, range 0.01–4.61; COX-1 median 0.45, range 0.17–3.51; COX-2 median 0.06, range 0.02–1.86; 15-PGDH median 0.04, range 0.00–2.17). In addition, the VAS pain was not lower in individuals who used NSAIDs (median 43, range 2–85) compared to those who did not (median 11, range 0–100).

**Fig 1:**
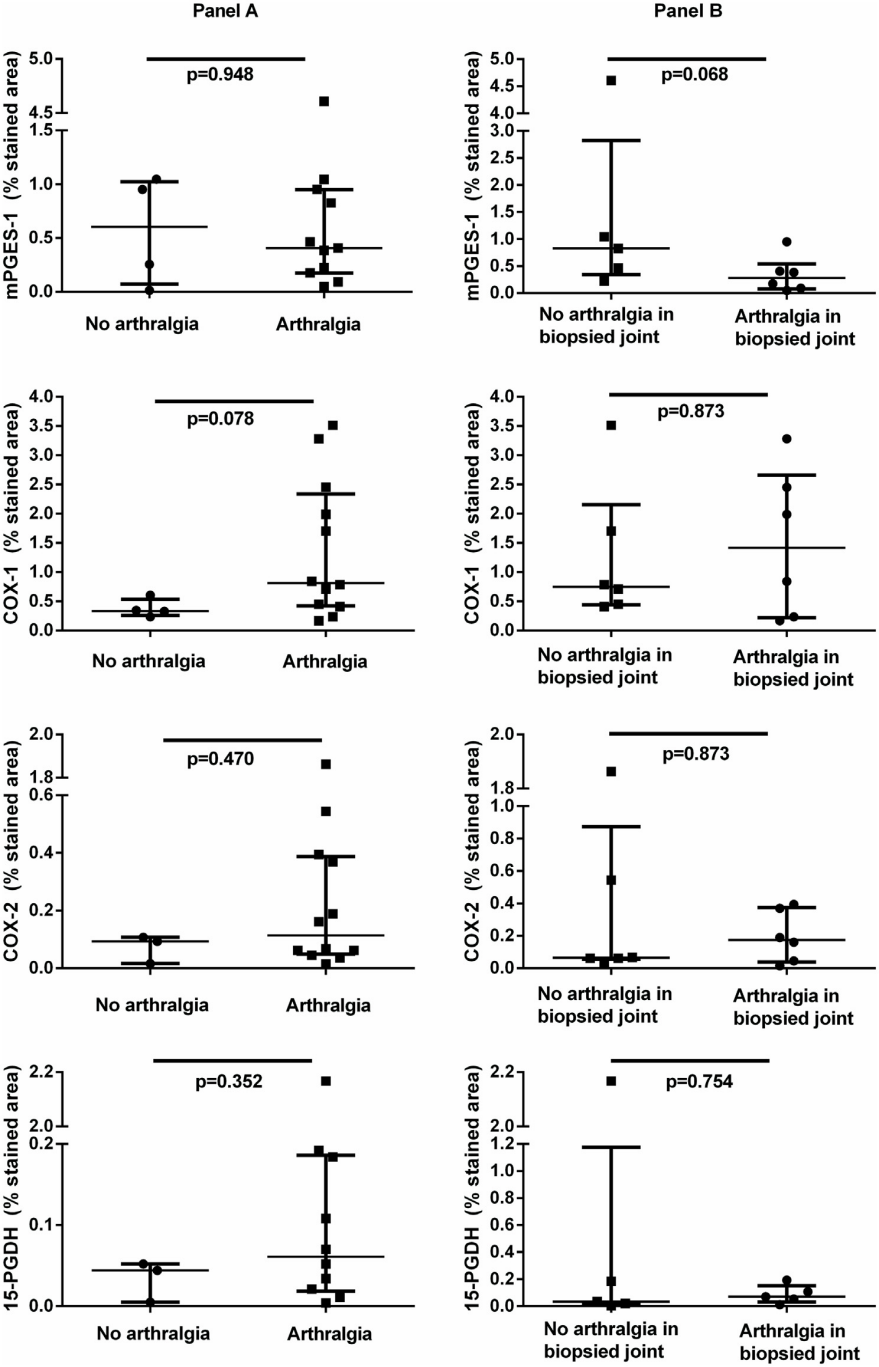
Synovial expression of mPGES-1, COX-1, COX-2 and 15-PGDH in autoantibody-positive individuals at risk of developing RA. Panel (A): comparison of individuals with or without arthralgia in general. Panel (B): comparison of individuals with or without arthralgia in the biopsied joint, within the group of individuals with arthralgia only; values expressed as median (IQR). mPGES-1: microsomal prostaglandin E synthase-1; COX: cyclooxygenase, 15-PGDH: 15-hydroxy prostaglandin dehydrogenase.

In 15 individuals with arthralgia compared to 4 individuals without arthralgia a trend towards higher expression of COX-1 was observed (p = 0.078) as well as COX-2 (p = 0.470) and 15-PGDH (p = 0.352) (Fig 1, panel A), which failed to reach statistical significance. Expression of PGE_2_ pathway enzymes did not correlate with pain scores (Table 2).

**Table 2:** Correlation analysis of pain scores and expression of PGE_2_ pathway enzymes in the synovium of autoantibody-positive individuals.

Enzyme	VAS pain general	68TJC	VAS pain biopsied joint
mPGES-1	r = 0.038; p = 0.892	r = -0.002; p = 0.995	r = -0.203; p = 0.467
COX-1	r = -0.141; p = 0.603	r = 0.112; p = 0.652	r = -0.126; p = 0.642
COX-2	r = -0.007; p = 0.980	r = 0.546; p = 0.035	r = -0.280; p = 0.313
15-PGDH	r = 0.029; p = 0.925	r = 0.211; p = 0.490	r = 0.037; p = 0.905

VAS: visual analogue scale; 68TJC: tender joint count of 68 joints; mPGES-1: microsomal prostaglandin E synthase-1; COX: cyclooxygenase; 15-PGDH: 15 prostaglandin dehydrogenase

Within the group of individuals with arthralgia, expression of PGE_2_ pathway enzymes was not higher in the individuals having arthralgia in the biopsied joint compared to the individuals having arthralgia in other joints only (Fig 1, panel B).

Next, we compared synovial expression of PGE_2_ pathway enzymes in individuals who developed arthritis (n = 6) with those who did not (n = 13), irrespective of their arthralgia status (one individual without arthralgia had developed arthritis after follow-up). We did not observe any differences in the expression levels of mPGES-1, COX-1, COX-2, or 15-PGDH between individuals who developed arthritis and those who did not (mPGES-1 median 0.32, range 0.09–0.83, vs 0.46, 0.01–4.61; COX-1 median 0.75, range 0.17–0.84, vs 0.53, 0.24–3.51; COX-2 median 0.11, range 0.06–0.37, vs 0.09, 0.02–1.86; 15-PGDH median 0.05, range 0.00–0.07, vs 0.05, 0.00–2.17). Collectively, these results suggest that synovial expression of the PGE_2_ pathway has no major role in either arthralgia or the onset of arthritis in autoantibody-positive individuals at risk of developing RA.

### Early arthritis patients

Hundred and four early arthritis patients were included in the study. Baseline characteristics of early arthritis patients are depicted in Table 3.

**Table 3:** Baseline characteristics of early arthritis patients.

	N = 104
Sex, female (n (%))	64 (62)
Age, years (mean (SD))	48 (15)
IgM-RF positive (n (%)) *	29 (29)
Anti-CCP positive (n (%))	27 (27)
IgM-RF and anti-CCP both pos (n (%)) *	22 (22)
ESR, mm/hr (median (IQR)	23 (11–45)
CRP, mg/L (median (IQR)	8.5 (3.2–27.4)
VAS pain general, mm (median (IQR))	61 (39–77)
VAS pain biopsied joint, mm (median (IQR))	59 (29–80)
DAS28 (median (IQR))	4.4 (3.1–5.6)
68 TJC (n)	5 (1–12)
66 SJC (n)	3 (1–8)
NSAID use (n (%)) **	57 (55)

IgM-RF = IgM rheumatoid factor; ACPA = anti-citrullinated protein antibodies; ESR = erythrocyte sedimentation rate; CRP = C-reactive protein; VAS = visual analogue scale (rang 0–100 mm); 68 TJC = tender joint count of 68 joints; 66 SJC = swollen joint count of 66 joints;

* missing for 3 patients;

** missing for 1 patient;

SD: standard deviation; IQR: interquartile range; NSAID: non steroidal anti-inflammatory drug

Fifty-five percent of the early arthritis patients in this study used NSAIDs at the moment of synovial biopsy sampling. Expression of mPGES-1, COX-1 and COX-2 was comparable between individuals who did or did not use NSAIDs (mPGES-1 p = 0.848, COX-1 p = 0.491, COX-2 p = 0.830). This suggests that NSAID treatment will not bias the results of our analyzes. In addition the VAS pain was comparable between individuals who did or did not use NSAIDs (p = 0.977).

#### Pain sensation is not related to synovial expression of PGE_2_ pathway enzymes in early arthritis patients

We did not find a positive correlation between synovial expression of PGE_2_ pathway enzymes and pain sensation in early arthritis patients (Table 4).

**Table 4:** Correlation analysis of pain scores and expression of PGE_2_ pathway enzymes in the synovium of early arthritis patients.

Enzyme	VAS pain general	68TJC	VAS pain biopsied joint
mPGES-1	r = -0.152; p = 0.137	r = -0.222; p = 0.025	r = -0.180; p = 0.088
COX-1	r = -0.083; p = 0.429	r = -0.336; p = 0.001	r = -0.187; p = 0.079
COX-2	r = 0.006; p = 0.953	r = -0.199; p = 0.053	r = -0.010; p = 0.928

VAS: visual analogue scale; 68TJC: tender joint count of 68 joints; mPGES-1: microsomal prostaglandin E synthase-1; COX: cyclooxygenase

Results were comparable when analyzing diagnostic subgroups of RA, SpA and UA (data not shown).

However, as expected since PGE_2_ is a mediator of vasodilation and oedema, we observed a positive correlation between expression of COX-1 and CD3 (T cells) and between expression of mPGES-1 and COX-1 and expression of CD68 (macrophages) both in the synovial sublining and lining layer (Table 5). None of the PGE_2_ pathway enzymes correlated to the systemic inflammatory parameters ESR and CRP, or to expression of CD55 (FLS) in the synovium.

**Table 5:** Correlation analysis of inflammatory markers and expression of PGE_2_ pathway enzymes in the synovium of early arthritis patients.

Enzyme	ESR	CRP	CD55 (N = 49)	CD3 (N = 47)	CD68SL (N = 45)	CD68L (N = 48)
mPGES-1	r = -0.057; p = 0.570	r = -0.044; p = 0.658	r = 0.277; p = 0.054	r = 0.258; p = 0.080	r = 0.359; p = 0.016	r = 0.326; p = 0.024
COX-1	r = 0.058; p = 0.566	r = -0.027; p = 0.789	r = 0.191; p = 0.188	r = 0.398; p = 0.006	r = 0.439; p = 0.003	r = 0.460; p = 0.001
COX-2	r = -0.040; p = 0.702	r = 0.009; p = 0.932	r = 0.117; p = 0.435	r = 0.272; p = 0.074	r = 0.180; p = 0.253	r = 0.203; p = 0.181

ESR: erythrocyte sedimentation rate; CRP: C-reactive protein; CD55: marker for fibroblast like synoviocytes; CD3: marker for T cells; CD68: marker for macrophages; SL: sublining layer; L: intimal lining layer; mPGES-1: microsomal prostaglandin E synthase-1; COX: cyclooxygenase

#### The expression of mPGES-1 and COX-1 is increased in SpA compared to RA patients

Baseline characteristics of the subgroups of early arthritis patients with a definite diagnostic classification of RA (n = 63), SpA (n = 14) or UA (n = 27) after 2 years of follow-up are depicted in Table 6. As expected, the number of patients being positive for IgM-RF and/or anti-CCP antibodies as well as the tender and swollen joint count and the DAS28 were significantly higher in the RA compared to the SpA and UA patients. The VAS for pain was significantly lower in SpA compared to UA and RA patients.

**Table 6:** Baseline characteristics of early arthritis patients classified as RA, SpA or UA after 2 years of follow-up.

	RA (N = 63)	SpA (N = 14)	UA (N = 27)	P-value
Sex, female (n (%))	43 (68)	5 (36)	16 (59)	0.074
Age, years (mean (SD))	51 (15)	44 (14)	44 (15)	0.062
IgM-RF positive (n (%))	28 (45) *	0 (0)	1 (4)**	0.000
Anti-CCP positive (n (%))	27 (44)*	0 (0)	0 (0)**	0.000
IgM-RF and anti-CCP both pos (n (%))	22 (36)	0 (0)	0 (0)	0.000
ESR, mm/hr (median (IQR)	24 (11–47)	17 (11–39)	22 (11–55)	0.277
CRP, mg/L (median (IQR)	7.2 (3.2–27.0)	9.0 (4.0–19.3)	11.0 (3.0–31.0)	0.771
VAS pain general, mm (median (IQR))	67 (45–78)	29 (18–57)	53 (36–76)	0.017
VAS pain biopsied joint, mm (median (IQR))	63 (34–81)	30 (17–73)	59 (30–76)	0.351
DAS28 (median (IQR))	4.7 (3.7–5.9)	3.8 (3.0–4.7)	3.9 (2.8–4.6)	0.020
68 TJC (n)	11 (4–22)	3 (1–5)	1 (1–3)	0.000
66 SJC (n)	6 (2–11)	1 (1–3)	1 (1–2)	0.000
NSAID use (n (%)) *	36 (57)	16 (62)	5 (36)	0.263

IgM-RF = IgM rheumatoid factor; anti-CCP = anti-cyclic citrullinated peptide antibodies; ESR = erythrocyte sedimentation rate; CRP = C-reactive protein; VAS = visual analogue scale (rang 0–100 mm); 68 TJC = tender joint count of 68 joints; 66 SJC = swollen joint count of 66 joints;

* missing for 1 patient;

** missing for 2 patients;

SD: standard deviation; IQR: interquartile range; NSAID: non steroidal anti-inflammatory drug

**Fig 2:**
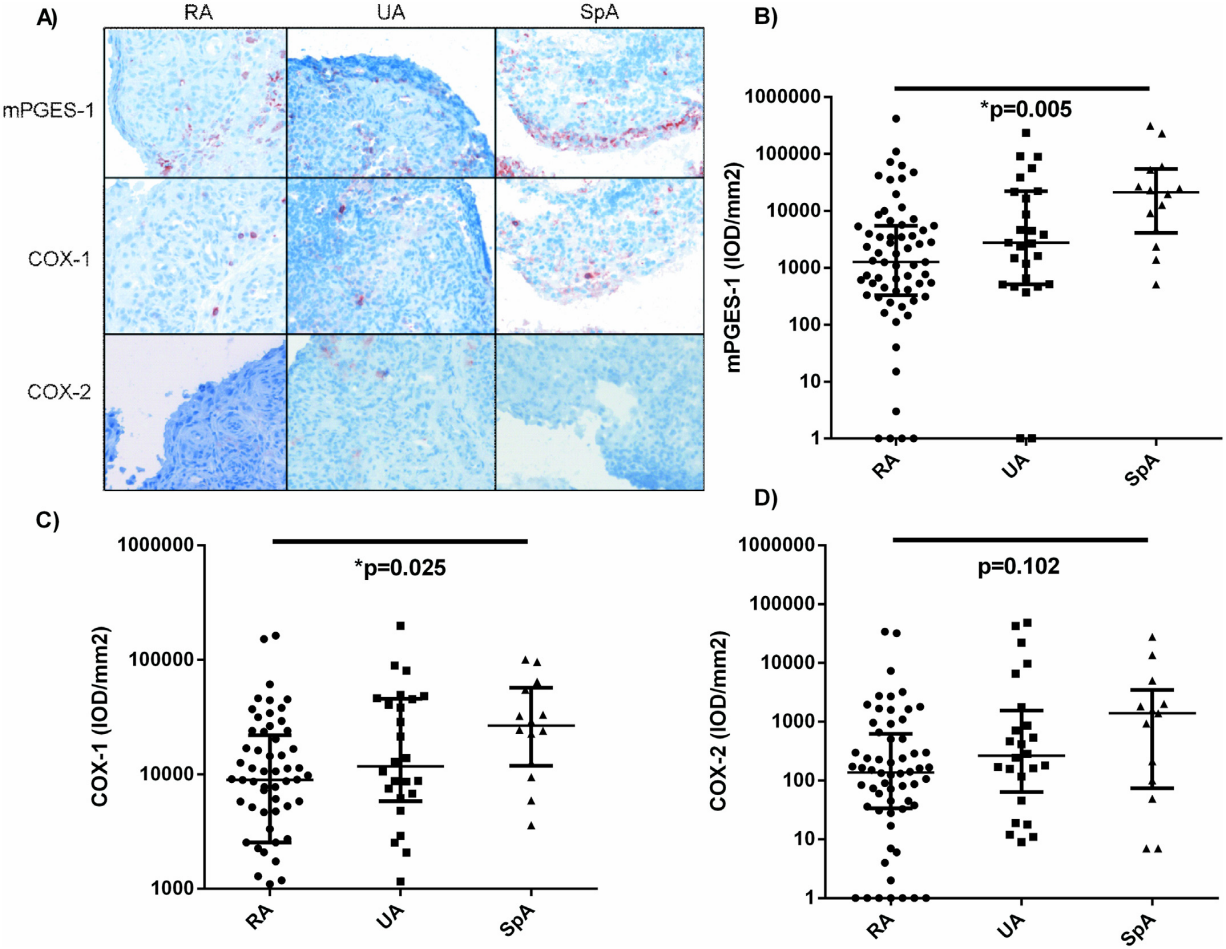
Baseline synovial expression of PGE2 pathway enzymes of early arthritis patients classified as RA, UA and SpA at 2 years follow-up. Representative immunohistochemical staining (A), Expression of mPGES-1 (B) and COX-1 (C) is increased in SpA patients. D) A trend towards increased expression of COX-2 is observed in SpA patients; values expressed as median (IQR) on a logaritmic scale; RA: rheumatoid arthritis; UA: unclassified arthritis; SpA: spondyloarthritis; mPGES-1: microsomal prostaglandin E synthase-1; COX: cyclooxygenase; IOD: integrated optical density.

Expression of mPGES-1,COX-1 and COX-2 was significantly different between the three diagnostic groups (p = 0.005, p = 0.021 and p = 0.044, respectively) (Fig 2). Tukey’s post-hoc test revealed that expression of mPGES-1 and COX-1 was increased in SpA compared to RA patients (p = 0.005 and p = 0.025, respectively) and that COX-2 expression was higher in SpA than RA patients although not statistically significant (p = 0.102).

#### The expression of PGE_2_ pathway enzymes is comparable between diagnostic outcome groups within UA and RA patients

To evaluate the discriminative value of synovial tissue expression of PGE_2_ pathway enzymes between UA patients who later fulfill classification criteria for RA and those who remain UA, we compared patients with UA at baseline who were classified as RA after 2 years of follow-up (UA>RA; n = 14) with UA patients at baseline who were still classified as UA after 2 years of follow-up (UA>UA; n = 27) and compared those with patients classified as RA already at baseline (RA>RA; n = 49). No significant difference could be detected in baseline synovial expression of mPGES-1, COX-1 or COX-2 between the three diagnostic outcome groups (Table 7).

**Table 7:** Baseline expression of PGE_2_ pathway enzymes in the synovium of early arthritis patients classified as UA>UA, UA>RA or RA>RA at baseline>after 2 years of follow-up.

Enzyme	UA>UA (N = 27)	UA>RA (N = 14)	RA>RA (N = 49)	P-value
mPGES-1 (median (IQR))	3569; (603–27983)	857; (204–5870)	1202; (468–4701)	0.144
COX-1 (median (IQR))	11912; (5588–44704)	9766; (5476–17992)	8620; (1659–22644)	0.214
COX-2 (median (IQR))	254; (63–1414)	133; (42–763)	143; (32–636)	0.260

UA: unclassified arthritis; RA: rheumatoid arthritis; UA>UA both at baseline and after 2 years of follow-up classified as UA; UA>RA: classified as UA at baseline and as RA after 2 years of follow-up; RA>RA: classified as RA both at baseline and after 2 years of follow-up.

mPGES-1: microsomal prostaglandin E synthase-1; COX: cyclooxygenase; Values expressed as integrated optical density (IOD)/mm2; median (IQR: interquartile range)

#### Synovial expression of PGE_2_ pathway enzymes is not related to disease persistence

Lastly, we compared baseline expression of PGE_2_ enzymes between prognostic outcome groups after 2 years of follow-up to evaluate whether RA patients developing persistent non-erosive (n = 30) or erosive (n = 11) disease had higher baseline expression levels than patients with self-liming disease (n = 13). Prognostic outcome data were missing for 9 patients. No statistically significant differences between the prognostic outcome groups were observed (Table 8).

**Table 8:** Baseline synovial expression of PGE_2_ pathway enzymes in different prognostic outcome groups of patients classified as RA after 2 years of follow-up.

Enzyme	Self-limiting (N = 13)	Persistent non-erosive (N = 30)	Persistent erosive (N = 11)	P-value
mPGES-1 (median (IQR))	560; (133–2305)	1661; (262–5183)	3330; (373–60408)	0.239
COX-1 (median (IQR))	8570; (4149–21901)	5045; (1737–17078)	13063; (8541–42152)	0.124
COX-2 (median (IQR))	111; (15–1794)	201; (51–876)	73; (53–155)	0.385

mPGES-1: microsomal prostaglandin E synthase-1; COX: cyclooxygenase

Values expressed as integrated optical density (IOD)/mm2; median (IQR: interquartile range)

## Discussion

This is the first study examining the expression of enzymes of the PGE_2_ pathway in synovial tissue of autoantibody-positive individuals at risk of developing RA and in early arthritis patients. First of all, within the group of autoantibody-positive individuals we observed a trend towards increased expression of COX-1, COX-2 and 15-PGDH in the group of individuals with arthralgia compared to the group without arthralgia. The value of the statistical analysis for this comparison was limited due to the small number of autoantibody-positive individuals without arthralgia. When looking within the overall group of 19 autoantibody-positive individuals synovial expression of enzymes of the PGE_2_ pathway did not correlate to absolute pain scores and the levels were not higher in individuals with arthralgia in the biopsied joint compared to individuals having arthralgia in other joints only. In the group of early arthritis patients, which was markedly larger, we did not observe a correlation with pain scores either. Therefore, our obervations suggest that pain sensation in individuals at risk of developing RA and in early arthritis patients cannot be clearly explained by altered synovial expression of PGE_2_ pathway enzymes. PGE_2_ generates pain by increasing the sensitivity of nociceptive neurons to pain mediators such as bradykinin [31], resulting in hyperalgesia. More precisely, PGE_2_ molecules interact with E prostanoid (EP) receptors on sensory nerve endings to lower their threshold of activation for other stimuli. Sensitization can occur both peripherally, in primary afferent nociceptors of the joint, and centrally, in spinal cord neurons and dorsal root ganglia. Inflammatory mediators other than PGE_2_, such as bradykinin, histamine, adenosine triphosphate (ATP), and acetylcholine [32], and the pro-inflammatory cytokines tumor necrosis factor (TNF) and interleukin-6 (IL-6) may be involved in pain sensation [33]. Therefore, the intensity of pain may not be dependent on solely the PGE_2_ pathway, since other inflammatory markers trigger and/or sensitize nerves to pain as well and pain experience might be a resultant of pain induced by several pathways. Another explanation for our findings may be that PGE_2_ would be involved in regulating arthralgia centrally (via spinal release) or via release by circulating monocytes that mediate the acute response, but not locally in the synovium. A third explanation could perhaps be that the quantification of enzymes by immunohistochemistry is not accurate enough for correlation with pain scores. An alternative method would have been to analyze the actual concentration of PGE_2_ in the synovial tissues. This was, however not possible due to limitation of biopsy material, and a clear disadvantage of analysis of whole biopsy samples without morphology is that it does not allow for selection of the specific synovial tissue rather than sublining connective or adipose tissue which would have been as a source of bias, in particular in subjects with a very thin synovial layer as observed in individuals without arthritis. Previous work showing a highly significant relationship between cytokine levels evaluated by immunohistochemistry on the one hand and a score for pain on the other indicate that quantification of sections stained by immunohistochemistry can be used to detect correlations with pain [6]. Measuring urinary excretion of the major PGE_2_ metabolite could have provided information about the systemic involvement of the PGE_2_ pathway [34], but urine samples were not available and this analysis would not have provided insight into expression levels at the target tissue, which was the scope of the research described here. We also need to bear in mind that other prostanoids which have been shown to mediate pain hypersensitivity could be involved. For example, studies in experimental models of arthritis have demonstrated involvement of PGI_2_ (prostacyclin) in arthritic pain [35].

The synovial expression of enzymes of the PGE_2_ pathway was not related to development of arthritis in subjects at risk of developing RA and within RA patients we did not observe a relation with the systemic inflammatory parameters ESR and CRP or with disease persistence. However, expression of these enzymes was positively correlated to the number of macrophages present in the synovium, which has been shown to be one of the main cell types expressing these enzymes [18].

Interestingly, in early arthritis patients, synovial expression of mPGES-1 and COX-1 was increased in SpA patients compared to RA and UA patients and a similar trend was observed for COX-2, suggesting involvement of the PGE_2_ pathway in the pathogenesis of SpA, supporting previous studies [17,36]. The SpA patients in our cohort displayed significantly lower pain scores than RA and UA patients. Previously, enhanced expression of COX-2 was observed in the intimal lining layer of ankylosing spondylitis (AS) patients (axial SpA) compared to psoriatic arthritis (PsA) and RA patients, and in the synovial sublining layer in AS and PsA patients compared to RA patients [17]. In that study, however, it could not be excluded that lower expression of COX-2 in the RA patient group was the result of the use of corticosteroids. In the current study, no patients had used corticosteroids for joint complaints or for any other disease in the last 3 months before inclusion, hereby excluding a treatment effect.

The fact that we found increased synovial expression of mPGES-1 and COX-1 in early SpA patients compared to early RA and UA patients may partly explain the difference in radiographic characteristics between RA and UA on the one hand (joint space narrowing and bone erosions) compared to SpA on the other (new bone formation). PGE2 can stimulate new bone formation by stimulating the differentiation of osteoblasts [35]. The beneficial effect of NSAIDs on the ossification of the spine in this patient group has repeatedly been shown [37]. Our results suggest that this might be linked to the presence of PGE_2_ produced in the synovial tissue. Moreover, a role for the PGE_2_ pathway in the pathogenesis of SpA has been shown by the positive effects of the use of NSAIDs, the first-line drug treatment for complaints of pain and stiffness in AS patients [38] and in SpA patients in general.

## Conclusions

In conclusion, we did not find clear evidence that arthralgia in subjects at risk of developing RA or in early arthritis patients can be explained by altered synovial expression of enzymes of the PGE_2_ pathway, which are known to be involved in pain sensation. Pain in these patients may therefore be regulated by other pathways or originate at sites other than the synovium, which needs to be addressed in future studies. Synovial expression of mPGES-1 and COX-1 is increased in SpA patients compared to RA and UA patients, supporting involvement of the PGE_2_ pathway in the pathogenesis of SpA.
